# Estimation of Multi-Species Leaf Area Index Based on Chinese GF-1 Satellite Data Using Look-Up Table and Gaussian Process Regression Methods

**DOI:** 10.3390/s20092460

**Published:** 2020-04-26

**Authors:** Yangyang Zhang, Jian Yang, Xiuguo Liu, Lin Du, Shuo Shi, Jia Sun, Biwu Chen

**Affiliations:** 1 School of Geography and Information Engineering, China University of Geosciences, Wuhan 430074, China; zhangyangyang@cug.edu.cn (Y.Z.); liuxiuguo@cug.edu.cn (X.L.); dulin@cug.edu.cn (L.D.); sunjia@whu.edu.cn (J.S.); 2 Artificial Intelligence School, Wuchang University of Technology, Wuhan 430223, China; 3 College of Resources and Environmental Sciences, Gansu Agricultural University, Lanzhou 730070, China; 4 State Key Laboratory of Information Engineering in Surveying, Mapping and Remote Sensing, Wuhan University, Wuhan 430079, China; shishuo@whu.edu.cn (S.S.); cbw_think@whu.edu.cn (B.C.)

**Keywords:** leaf area index (LAI), look-up table (LUT), Gaussian process regression (GPR), GF-1, PROSAIL

## Abstract

Leaf area index (LAI) is an important biophysical parameter, which can be effectively applied in the estimation of vegetation growth status. At present, amounts of studies just focused on the LAI estimation of a single plant type, while plant types are usually mixed rather than single distribution. In this study, the suitability of GF-1 data for multi-species LAI estimation was evaluated by using Gaussian process regression (GPR), and a look-up table (LUT) combined with a PROSAIL radiative transfer model. Then, the performance of the LUT and GPR for multi-species LAI estimation was analyzed in term of 15 different band combinations and 10 published vegetation indices (VIs). Lastly, the effect of the different band combinations and published VIs on the accuracy of LAI estimation was discussed. The results indicated that GF-1 data exhibited a good potential for multi-species LAI retrieval. Then, GPR exhibited better performance than that of LUT for multi-species LAI estimation. What is more, modified soil adjusted vegetation index (MSAVI) was selected based on the GPR algorithm for multi-species LAI estimation with a lower root mean squared error (RMSE = 0.6448 m^2^/m^2^) compared to other band combinations and VIs. Then, this study can provide guidance for multi-species LAI estimation.

## 1. Introduction

The leaf area index (LAI) is usually defined as one half of the total green leaf area per unit of horizontal ground surface area [1]. As the key vegetation biophysical parameter, LAI can be efficiently applied in monitoring vegetation photosynthesis, respiration, transpiration, soil respiration, and the energy exchange of the canopy-atmosphere [2,3]. Hence, LAI has been generally applied in many fields, such as precision agriculture, vegetation health monitoring, and crop yield estimation [4,5,6,7]. Therefore, it is significant to accurately invert vegetation LAI in real time. At present, the field measurement methods of LAI mainly contain the direct destructive sampling method, the indirect digital hemispherical photography method, plant canopy analyzers, and smartphones application named PocketLAI [8]. However, the classical methods are difficult to obtain LAI for large areas. In addition, crop LAI is also difficult to obtain by field measurements due to the high spatial and temporal heterogeneity.

Remote sensing techniques provide an effective method for quantitative assessment of LAI due to the advantage of fast, non-destructive and large scales. Many studies have successfully retrieved LAI through using remote sensing data [9,10,11,12,13,14,15]. The LAI inversion methods by remote sensing data are generally divided into two main aspects: statistical and physical approaches [16,17,18]. The statistical approaches are usually in term of the linear or nonlinear relationship between ground-based LAI measurements and spectral vegetation indices [19,20], which are simple and fast. However, a certain physical mechanism and universality is lacking [21,22]. The physical inversion strategies are built based on the radiative transfer models (RTM) [23,24]. There are three different inversion strategies for LAI estimation: iterative optimization, look-up table (LUT), and hybrid inversion [25,26]. The iterative optimization is a classical inversion approach, but it needs high computational cost and exhibits poor convergence. Additionally, many studies have shown that the LUT and hybrid inversion exhibited better performance for LAI retrieval [27,28].

The LUT relies on a large multiple-solutions database (rather than the single best solution) for the inversion of vegetation parameters. Measurements or model errors have little influence on this inversion method [29], which improved the robustness of LAI inversion [30], the LUT method has been widely used for LAI inversion in many studies due to the advantage of stability and robustness [31,32,33]. Hybrid inversion, which was combined the RTM with machine learning regression algorithms (MLRAs), uses RTM to generate simulated datasets as the training set of MLRAs. The hybrid inversion has the advantages of the statistical approaches’ simplicity and the physical method’s universality, which can accurately and rapidly invert vegetation LAI [34,35]. All kind of MLRAs are applied for the inversion of RTM, for instance, artificial neural network (ANN), support vector regression (SVR), random forest (RF), and Gaussian process regression (GPR) [36,37,38,39]. Several studies have shown that GPR exhibits more performance for LAI estimation among these MLRAs [36,40,41].

Using a high-resolution satellite is an effective way to extract LAI information, which can better meet the requirements for monitoring geographical conditions. Thus, the China High Resolution Earth Observation System (CHEOS) affords high spatial–temporal and near-real-time (NRT) observation information for vegetation estimation. Chinese satellites of High-Resolution Satellite 1 (GF-1) with a swath width of 800 km, coverage repetitive cycle of 4 days, spatial resolution of 16 m, the first satellite of CHEOS, was launched on 26 April 2013. In this study, GF-1 data was used to invert vegetation LAI and generate the LAI map.

With the development of remote sensing techniques, many studies have been conducted on LAI inversion based on remote sensing data. However, most of these studies may be only focused on the single species LAI estimation, such as wheat [32,33,42,43], maize [44,45], and sugar beet [26,46]. Relatively few research has been carried out on LAI inversion of multi-species. However, plant types are mostly mixed rather than single distribution. It is worth exploring whether the LAI of multi-species can be rapidly and efficiently retrieved based on remote sensing data.

Therefore, the objectives of this study are to (1) evaluate the suitability of GF-1 data for multi-species LAI estimation by using the LUT and GPR algorithms combined with the PROSAIL RTM; (2) explore the performance of the LUT and GPR for estimating multi-species LAI in term of 15 different band combinations and ten published vegetation indices (VIs); and (3) analyze the effect of the different band combinations and VIs on the accuracy of LAI estimation.

## 2. Study Area and Data

### 2.1. Study Area

**Figure 1:**
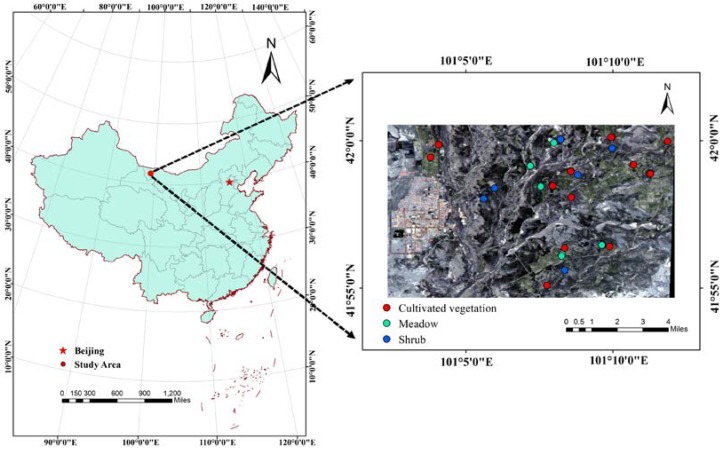
Location of the study area and spatial distribution of the sample plots.

Heihe River Basin of China is located in the middle of the Hexi Corridor (96°42′–102°00′E, 37°41′–42°42′N), which is the second largest inland river basin in the northwest China. Heihe River Basin is located in the center of Eurasia, which is far away from the ocean and surrounded by mountains. The climate of the basin is mainly controlled by westerly circulation in mid-high latitude and is influenced by cold air mass in the polar regions. Thus, it has a typical continental climate, the precipitation is rare and concentrated, the sunshine is sufficient and the temperature difference between day and night is large. In this study, the lower reaches of the Heihe River Basin are selected as the study area (Figure 1). The study area includes a large of the Gobi Desert, which contains desert riparian forests, shrub, and meadow vegetation [47].

### 2.2. Field Measurement Data

As the study area is located in the northwest of China, the average temperature and precipitation in July will reach the maximum value, and vegetation grows vigorously, which is conducive to the measurement and evaluation of LAI. Then, field LAI observation started from 22 July and ended on 1 August in 2014. Considering the characteristics of downstream vegetation distribution, uniform vegetation with large area distribution (over 100 m × 100 m) was selected as the observation sample plot. A total of 29 observation sample plots were selected, and the plant species included Hami melon, *Tamarix chinensis*, reed, weed, flower wood, bitter bean, and so on (Figure 1). LAI field observations were non-destructively measured by using the LAI-2200 plant canopy analyzer (LI-COR, Inc., Lincoln, NE, USA). The average LAI was obtained based on one above-canopy measurement and four below-canopy measurements of the incoming radiation in each sample plot, every sample plot was repeatedly measured at least 2 times, and the measurement time of all samples was from 8:00 to 12:00 [48]. Due to the experimental conditions limited, the data of the field measurements were downloaded from the Cold and Arid Regions Science Data Center. Then, every plot was matched with GF-1 pixel by using the ENVI 5.3 software according to the longitude and latitude coordinates.

### 2.3. Remote Sensing Data

GF-1 Wild Field Camera (WFV) data was used to estimate LAI of the study area. GF-1 was launched by China on 26 April 2013, and the GF-1 WFV camera can obtain 16 m multi-spectral color images (Blue, Green, Red, and Near-infrared bands). GF-1 satellite is equipped with four WFV camera sensors, namely WFV1, WFV2, WFV3, and WFV4, which simultaneously image and have different absolute radiometric calibration coefficients in orbit. The imaging swath width of each WFV sensor is about 200 km, and the imaging swath width of the four WFV sensors can reach about 800 km. The key parameters of the WFV sensor were listed in Table 1. Due to the existence of clouds or haze, a high-quality GF-1 WFV image data on 29 July in 2014, which was covered in the study area, was downloaded.

To convert the digital number (DN) of the raw image to land surface reflectance, the ENVI 5.3 software was used to preprocess the GF-1 image data in this study, including radiometric calibration, atmospheric correction, and geometric correction.

## 3. Methods

### 3.1. Inversion Schemes

**Figure 2:**
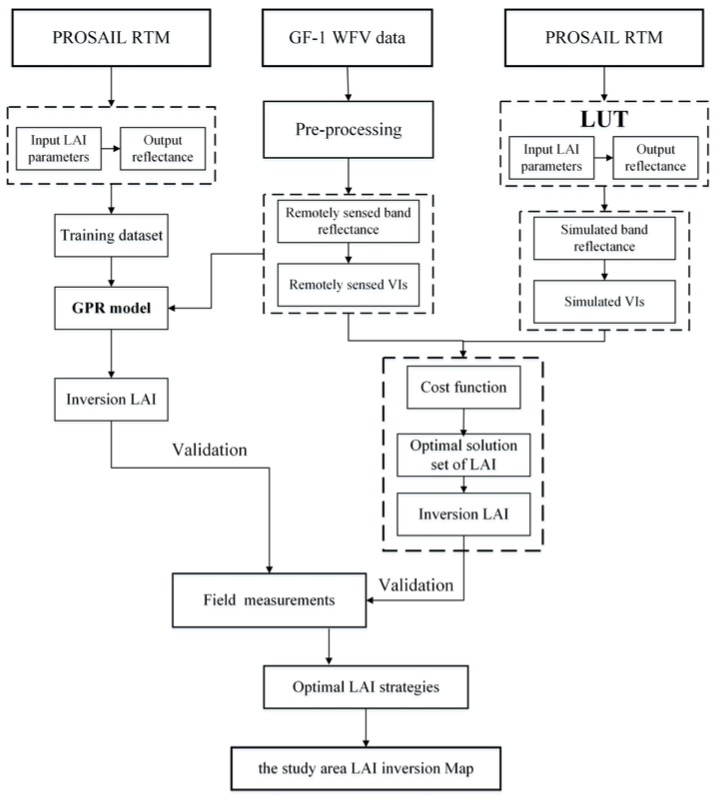
Flow chart of leaf area index (LAI) inversion for this study.

Figure 2 shows the inversion flow chart of this study. Firstly, LAI was evaluated based on the reflectance of GF-1 WFV and VIs. In order to fully assess the performance of the spectral information for LAI estimation, 15 combinations of four bands were used (Table 2). In addition, LAI and VIs had a good correlation according to previous studies, so 10 types of VIs were selected, which were listed in Table 3. These VIs, which included simple difference indices, a normalized difference ratio, triangular vegetation indices, and modified versions, were commonly used to estimate LAI [19,31,33]. The relative wavelengths of VIs were located in the visible and near-infrared bands and in the GF-1 band ranges. Then, LUT and GPR methods were adopted to realize LAI inversion based on remote sensing spectral bands and VIs by using PROSAIL model. Finally, the optimal strategies were achieved through the verification of ground measurements data. 

### 3.2. LUT Inversion

The LUT inversion is a conceptually simple approach to retrieve LAI based on a large multi-solution database. Thus, the database determines the implementation of LUT. Generally, the simulated dataset has been generated by PROSAIL RTM, which is a combination of the SAIL (scattering by arbitrarily inclined leaves) [60] canopy reflectance model and PROSPECT leaf optical properties model [26]. In this study, PROSAIL5B, a combination of PROSPECT5 and 4SAIL, was used to simulate canopy reflectance with many input canopy biophysical parameters through the output leaf reflectance and transmittance of PROSPECT with some input leaf biochemical parameters to SAIL [61]. To build LUT, the PROSAIL model was used to simulate the broadband canopy reflectance of GF-1 WFV through randomly generated input parameters. The key input parameters of PROSAIL are summarized by many literatures, which was listed in Table 4 [33,62,63,64,65]. Due to the lack of understanding of the actual growth status of vegetation in the study area, the simulation dataset should contain enough vegetation types and states to improve the robust of LUT and GPR for LAI estimation. In addition, the brown pigment of the PROSPECT model was also set. To get appropriate inversion results, Weiss et al. found that the size of LUT needs 100,000 reflectance/VIs, which can well balance the contradiction between saving computing resources and improving inversion accuracy [27]. 

To select the solution of the inverse problem, the LUT solution can be calculated by averaging similar parameter combinations with the smallest differences between the reflectance/VIs and the simulated reflectance/VIs [61,66,67]. The relative root mean square error (RRMSE) was used as the cost function to assess the performance of model for LAI estimation in this study, and was defined as follows:

(1) $$RRMSE_{R}=\sqrt{\frac{1}{m}\sum_{i=1}^{m}{\left(\frac{R_{RS,i}-R_{Simulated,i}}{R_{RS,i}}\right)}^{2}}$$

(2) $$RRMSE_{VI}=\sqrt{{\left(\frac{VI_{RS,i}-VI_{Simulated,i}}{VI_{RS,i}}\right)}^{2}}$$

where *R_RS_* is the measured band reflectance; *R_Simulated_* is the simulated reflectance; *VI_RS_* is the measured *VI*; *VI_Simulated_* is the simulated *VI*, which was calculated from the simulated reflectance; and *m* is the number of spectral bands.

A set of reflectance/VIs variables corresponding to the minimization of the RRMSE value in the LUT are taken as the solution. However, the solution may not be the only and optimal result due to measurement errors and model deficiencies. The solution selected a threshold 10% of the ordered minimum RRMSE and these parameter combinations were averaged with the LUT size of 100,000, which can solve the ill-posed inversion problem. It was feasible for the accuracy of LAI estimation with the 10% threshold, and was consistent with the previous studies [61,67].

### 3.3. GPR Inversion

GPR is a nonparametric model for regression analysis of data using the Gaussian process prior. The model hypothesis of GPR includes two parts: noise (regression residual) and Gaussian process prior, and the essence of its solution is Bayesian inference. Without limiting the form of kernel function, GPR is theoretically a universal approximation of any continuous function on a compact space. GPR uses the Gaussian process as a priority, which assumes that the training samples are the sampling of Gaussian process, which resulted in estimation results are closely related to the kernel function. The practical significance of the kernel function in GPR is a covariance function, which describes the correlation among training samples. In this study, a squared exponential kernel function was applied in the GPR model, which was a widely used standard covariance function. Moreover, the squared exponential kernel function can capture sample similarity well in most problems and just one hyperparameter needed to be tuned. The detailed introduction of GPR and kernel functions can be referred to in [36,68,69,70].

In this paper, GPR inversion uses the PROSAIL model to build a simulated dataset that including simulated spectral reflectance/VIs and corresponding simulated LAI as the training dataset, to obtain the regression relationship between spectral reflectance/VIs and LAI. This inversion method has the advantages of simple empirical methods and strong university of physical model, and can accurately and rapidly retrieve LAI. The number of GPR training datasets would be determined in the next part.

### 3.4. Statistical Evaluation 

To evaluate the performance of LUT and GPR for LAI estimation, the coefficient of determination (R^2^) and root-mean-square error (RMSE) were used. Sets $y_{i}$ and $y_{i}^{’}$ denote the measured and predicted values respectively, $\bar{y}$ refers to the average of $y_{i}$, and n is the number of measurements:

(3) $$R^{2}=1-\frac{\sum_{i=1}^{n}{\left(y_{i}-{y_{i}}^{\prime }\right)}^{2}}{\sum_{i=1}^{n}{\left(y_{i}-\bar{y}\right)}^{2}}$$

(4) $$RMSE=\sqrt{\frac{\sum_{i=1}^{n}{\left(y_{i}-{y_{i}}^{\prime }\right)}^{2}}{n}}$$

## 4. Results 

### 4.1. Determination of the Number of GPR Training Datasets

The synthetic dataset generated by the PROSAIL model was used as the training dataset of GPR model, which solved the demand of the model for the measured dataset. The training dataset directly influences the inversion accuracy of the GPR model. However, the training dataset is not positively correlated with the inversion accuracy of the model. This possible interpretation is that the accumulation of the random Gaussian noise and the occurred of the overfitting of the training model with the increasing of the training dataset number may be influenced by the accuracy of the LAI estimation. In this study, a different number of training datasets was used to analyze the accuracy of LAI inversion based on GPR.

**Figure 3:**
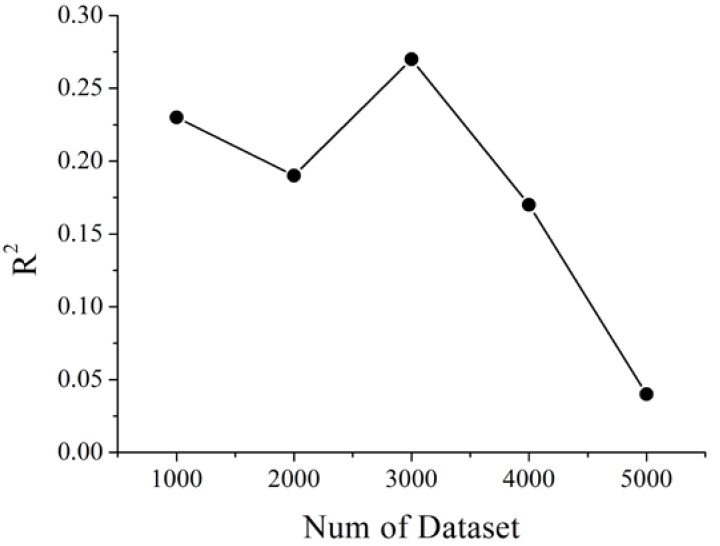
Accuracy of LAI inversion accuracy with a different number of training datasets based on Gaussian process regression (GPR).

As it can be seen from Figure 3, when the number of training datasets was 3000, the inversion accuracy of GPR was optimal. García-Haro, F. J. also introduced that when the number of training samples by PROSAIL model generation was around 3000, the RMSE values of LAI estimation becomes stable [65]. Therefore, 3000 simulated datasets were selected as training datasets of GPR model in this paper.

### 4.2. Different Band Combinations on LAI Inversion

**Figure 4:**
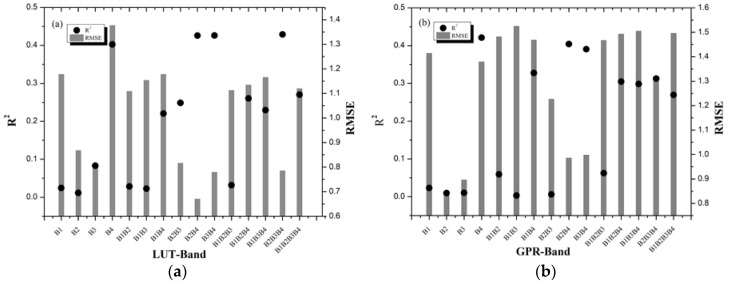
Comparison of LUT and GPR with different bands of reflectance to predict LAI based on GF-1 data ((**a**) LUT and (**b**) GPR).

The performance of LUT and GPR for LAI inversion with different band combinations (Table 2) was evaluated and was shown in Figure 4. Theoretically, the best inversion strategy is to have the highest R^2^ and the lowest RMSE, but in most cases, R^2^ and RMSE are not always consistent. For the LAI estimation based on the LUT-Band strategies with the band reflectance of GF-1 data (Figure 4a), the B2B4 (R^2^ = 0.4258, RMSE = 0.6700 m^2^/m^2^) exhibits the optimal performance for LAI inversion by the comparison of R^2^ and RMSE values, which was selected to estimate LAI in the following study. In addition, the B3B4 (red, Nir; R^2^ = 0.4260, RMSE = 0.7786 m^2^/m^2^) and B2B3B4 (green, red, Nir; R^2^ = 0.4290, RMSE = 0.7848 m^2^/m^2^) also displayed the potential for predicting the LAI based on GF-1 data. The remaining LUT-Band had a relatively lower RMSE values (RMSE < 1.2 m^2^/m^2^), but the R^2^ values of LAI inversion were lower than 0.3. However, the R^2^ and RMSE of the B4 (Nir; R^2^ = 0.4026, RMSE = 1.3767 m^2^/m^2^) performed very inconsistently.

The GPR-Band strategies based on GF-1 data (Figure 4b) showed an inconsistency in R^2^ and RMSE. The B2 (green) and B3 (red) strategies had the lowest RMSE values (0.8470 m^2^/m^2^ and 0.8963 m^2^/m^2^) but also had the worst R^2^ values (0.0096 and 0.0102). The B2B4 (Green, Nir) and B3B4 (Red, Nir) of GPR performed consistently with a lower RMSE values (0.9856 m^2^/m^2^ and 0.9975 m^2^/m^2^) and a higher R^2^ values (0.4042 and 0.3906). The B4 (Nir) of GPR on LAI estimation appeared to be similar to the LUT-B4, with the highest R^2^ value of 0.4212 and a higher RMSE value of 1.3792 m^2^/m^2^. All the other GPR-Band strategies on LAI inversion performed poor accuracy with all the R^2^ lower than 0.3.

These results indicated that it is important to select the appropriate band combination strategy to improve the accuracy of LAI estimation by using LUT and GPR methods. Thus, the optimal inversion strategy should be selected by comprehensive consideration of R^2^ and RMSE. For LUT and GPR, the B2B4 (green, Nir) strategy exhibited the optimal performance for LAI estimation. However, the GPR took only one-third of the time of LUT. Thus, the B2B4 was selected as the spectral characteristics parameter to analyze LAI by using the GPR model.

### 4.3. Different Published VIs on LAI Estimation

**Figure 5:**
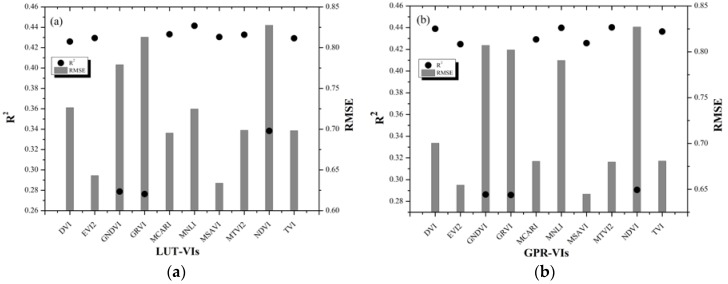
Comparison of LUT and GPR with different VIs to estimate LAI based on GF-1 data ((**a**) LUT and (**b**) GPR).

The performance of LUT and GPR for LAI inversion with different VIs (Table 3) was evaluated and was shown in Figure 5. For LUT and GPR, ten published VIs exhibited the basically similar performance for LAI estimation. Modified soil adjusted vegetation index (MSAVI) strategy got the best accuracy of LAI inversion based on the GF-1 data in both LUT and GPR with the low RMSE values (0.6338 m^2^/m^2^ and 0.6448 m^2^/m^2^) and a high R^2^ (0.4303 and 0.4257). In particular, the green normalized difference vegetation index (GNDVI), green ratio vegetation index (GRVI), and normalized difference vegetation index (NDVI) strategies all achieved a poor accuracy with a lower R^2^ and a higher RMSE for both LUT and GPR. Apart from the above VIs, the remaining VIs of LUT and GPR performed moderately.

Figure 5 shows that different VIs could achieve different inversion results for LUT and GPR methods. For the comparison of the R^2^ and RMSE values of LAI estimation based on different published VIs, MSAVI was considered as the optimal strategy to estimate multi-species LAI based on the GF-1 satellite data and selected for LAI estimation in the following study.

### 4.4. Comparison of LUT and GPR with Different Inversion Strategies on LAI Estimation

In this study, the suitability of GF-1 data for multi-species LAI estimation was discussed by using LUT and GPR algorithms based on the PROSAIL RTM. The accuracy of VIs-based inversion was higher than that of band-based inversion by using LUT. MSAVI exhibited the optimal performance for LAI estimation with the low RMSE value of 0.6338 m^2^/m^2^ and the relatively high R^2^ value of 0.4304 (Figure 4a and Figure 5a). Similar to the LUT inversion results, the VIs strategies also performed better performance for the LAI estimation than that of the band-based by using GPR. Additionally, MSAVI still achieved the optimal performance based on GPR with the low RMSE value of 0.6448 m^2^/m^2^ and the relatively high R^2^ value of 0.4257 (Figure 4b and Figure 5b). The reason of why the VIs inversion results was superior to that of the band-based may be that the VIs not only included sensitive bands of LAI, and also strengthened the difference among the reflectivity of bands, and weakened the influences of some factors (soil background). In addition, it is significant to choose an appropriate inversion strategy than an inversion method in order to improve the accuracy of LAI inversion. According to the accuracy of LAI inversion, the MSAVI is the optimal VIs by using LUT and GPR. However, GPR takes only one-third of the time of LUT regardless of the inversion strategy. Thus, considering the factors of precision and efficiency, MSAVI based on GPR was select as the optimal inversion method to estimate multi-species LAI from GF-1 satellite data in this study.

### 4.5. Mapping of Multi-Species LAI

Based on the above comparative analysis results, MSAVI based on GPR algorithm was selected for multi-species LAI retrieval by the GF-1 WFV data.

**Figure 6:**
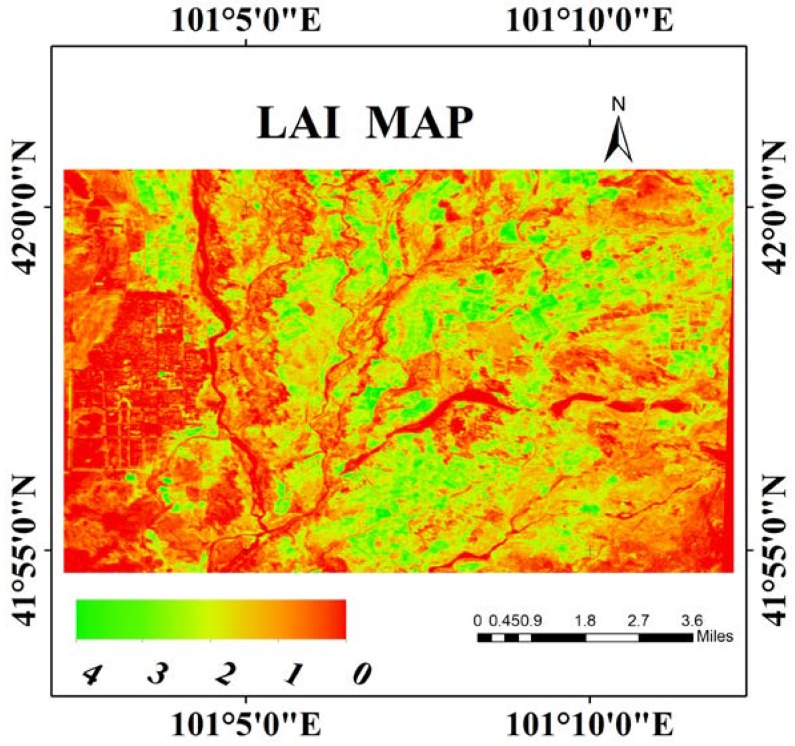
LAI map of the Heihe study area estimated by the modified soil adjusted vegetation index (MSAVI) strategy of GPR based on the measured GF-1 data on 29 July, 2014.

Figure 6 shows the LAI map generated from MSAVI strategy of GPR based on GF-1 data on 29 July, 2014. The LAI inversion values of the study area ranged from 0 to 4. Since the study area included a large area of the Gobi Desert, LAI values of the whole region were all low. The low LAI values were distributed in the west of the study area, and the high LAI values mostly appeared in the middle region. In general, the LAI retrieval map was basically consistent with the actual situations, and there was good consistency between the inversion and measured LAI values with the RMSE value of 0.6448 m^2^/m^2^.

## 5. Discussion

Multi-species LAI (e.g., Hami melon, *Tamarix chinensis*, reed, weed, flower wood, bitter bean, and so on) obtained from the GF-1 WFV sensor data was evaluated by comparing the different bands and VIs strategies based on the LUT and GPR methods. The optimal inversion strategy and method for multi-species LAI retrieval were determined. Additionally, the LAI map with the RMSE of 0.6448 m^2^/m^2^ was finally acquired through the optimal inversion strategy. Compared with the previous studies [71,72,73], the optimal inversion strategy exhibited better performance for multi-species LAI estimation. Thus, the GF-1 WFV data has good potential being used for multi-species LAI retrieval by choosing the appropriate inversion strategy. However, there are still some issues that need to be solved.

The inversion accuracy of LAI by using LUT is generally similar to that of GPR, which is in agreement with the previous studies [27,28]. However, the calculative process of LUT is slower and more complex than that of GPR, and some initial values of parameters also need to be set. Additionally, GPR is a hybrid inversion approach based on the RTM, which is different from the empirical method and does not need lots of measured data. The hybrid approach only requires measured data to verify the inversion accuracy. Compared with other inversion methods based on RTM, the hybrid inversion model has advantages in simple, fast, and accurate calculation in estimating vegetation parameters [35,74].

Different inversion strategies have a great important influence on the accuracy of LAI estimation. Fifteen band combinations and 10 VIs strategies of LUT and GPR displayed different performance for LAI estimation. In particular, B2B4 and MSAVI are the optimal methods on LAI inversion by GF-1 data. Both MSAVI and B2B4 strategies contain a near-infrared wavelength, which indicates that the near-infrared band of the GF-1 WFV sensor is sensitive to LAI estimation with multi-species on the underlying surface. It provides a reference for LAI inversion under similar underlying surface using the GF-1 WFV sensor data in the future study.

Due to the variety of plant types that was included in the study area, the improvement of LAI inversion accuracy is limited to a certain extent. The possible interpretation is that LAI of different plant types presents different special characteristics [75,76], as well as the application of inversion strategies. At present, many studies’ main focus on estimating LAI for a single plant type [3,14,32,33,43,53,67], while relatively few studies also discussed LAI estimation from multiple plant types [71,72,73]. These studies estimated global LAI by using different inversion strategies based on Sentinel-2 satellite data. The performance of Sentinel-2-derived for LAI estimation was quantified using global ground observation with consistent measurement criteria [73], and the RMSE value of Sentinel-2 LAI was 1.09 m^2^/m^2^ through comparison to ground LAI. The Simplified Level 2 Product Processor (SL2P) was used to estimate multi-crop LAI by Sentinel-2/MSI and Landsat-8/OLI data and was validated by using an agricultural area [71]. Additionally, the RMSE for MSI/PL2P and OLI/SL2P were 0.98 m^2^/m^2^ and 1.63 m^2^/m^2^ for LAI estimation, respectively. A new simple Sentinel-2 LAI index (SeLI) was proposed and used for multi-crop green LAI estimation [72]. SeLI performed well and had the potential to be used for LAI estimation of a multi-crop dataset (RMSE = 0.69 m^2^/m^2^). Compared with previous studies, it is feasible to use GF-1 data for multi-species LAI estimation. To improve the LAI inversion accuracy of multiple plant types, an efficient use of prior knowledge and accurate classification of different plant types and other effective methods will be studied in future work. In addition, LAI estimation of different species is also need to be further discussed by using classification and clustering in the following work.

## 6. Conclusions

In this study, the performance of GF-1 data for multi-species LAI estimation by using LUT and GPR algorithms combined with the PROSAIL RTM was analyzed. The effect of the 15 different bands combinations and 10 published VIs on the accuracy of LAI estimation was also assessed. The results demonstrated that the accuracy of VIs-based inversion was higher than that of band-based inversion for LAI estimation by using LUT and GPR. Moreover, the MSAVI strategy got the optimal performance of LAI inversion based on GF-1 data in both LUT and GPR with a high R^2^ (0.4303 and 0.4257) and low RMSE values (0.6338 m^2^/m^2^ and 0.6448 m^2^/m^2^). Nevertheless, GNDVI based on LUT and GPR had the worst accuracy of LAI estimation with the RMSE of 0.7790 m^2^/m^2^ and 0.8070 m^2^/m^2^, and the R^2^ of 0.2788 and 0.2863, respectively. These results indicated that different inversion methods had a great influence on LAI estimation. Selecting an appropriate inversion model can effectively improve the accuracy of the LAI estimation. If just considering the R^2^ values, the performance of the LUT (R^2^ = 0.4303) may be similar to that of GPR (R^2^ = 0.4257) for LAI estimation based on MSAVI. However, the running time of GPR was only one-third of that of LUT. Thus, GPR combined with MSAVI was optimal and was selected for multi-species LAI estimation in term of the GF-1 data. This study suggested that GF-1 satellite data performed well and had the potential to be used for multi-species LAI estimation. In addition, it is of great significance and value to research multi-species LAI inversion by using remote sensing satellite data.

## Figures and Tables

**Table 1:** Key characteristics of GF-1 Wild Field Camera (WFV).

Band	Wavelength Range (nm)	Radiometric Resolution (bit)	Spatial Resolution (m)	Breadth (km)	Revisit Period (d)
Blue (Band 1)	450–520	10	16	200 (1 CCD) 800 (4 CCD)	4
Green (Band 2)	520–590				
Red (Band 3)	630–690				
Near-infrared (Band 4)	770–890				

**Table 2:** The 15 combinations of four bands of GF-1 WFV.

No.	Band	No.	Band	No.	Band
1	B1	6	B1, B3	11	B1, B2, B3
2	B2	7	B1, B4	12	B1, B2, B4
3	B3	8	B2, B3	13	B1, B3, B4
4	B4	9	B2, B4	14	B2, B3, B4
5	B1, B2	10	B3, B4	15	B1, B2, B3, B4

Note: B1, B2, B3, and B4 are the wavelengths of blue, green, red, and near-infrared, respectively.

**Table 3:** Selected ten vegetation indices (Vis) from previous studies.

No.	VI	Formula	Reference
1	NDVI	(B4 − B3)/(B4 + B3)	[49,50,51]
2	DVI	B4 − B3	[52]
3	TVI	0.5 (120 (B4 − B2) − 200 (B3 − B2))	[53]
4	EVI2	2.5(B4 − B3)/[(B4 + 2.4B3) + 1]	[54]
5	GNDVI	(B4 − B2)/(B4 + B2)	[55]
6	GRVI	B4/B2 − 1	[56]
7	MCARI	$\frac{1.5[2.5(B4-B3)-1.3(B4-B2)]}{\left(\sqrt{{\left(2B4+1\right)}^{2}-(6B4-5\sqrt{B3})}-0.5\right)}$	[57]
8	MNLI	1.5 (B42 − B3)/(B42 + B3 +0.5)	[58]
9	MSAVI	$\frac{(2B4+1)-\sqrt{{\left(2B4+1\right)}^{2}-8(B4-B3)}}{2}$	[59]
10	MTVI2	$\frac{1.5[(1.2(B4-B2)-2.5(B3-B2))]}{\sqrt{{\left(2B4+1\right)}^{2}-(6B4-5\sqrt{B3})-0.5}}$	[19]

**Table 4:** Ranges and distributions of PROSAIL input parameters for the look-up table (LUT) generation.

Parameter	Variables	Unit	Max	Min	Average	Std.	Type
Leaf	N	—	2.5	1	1.5	1	Gaussian
	Cab	μg.cm^-2^	90	0	50	40	Gaussian
	Car	μg.cm^-2^	20	0	10	7	Gaussian
	Cbrown	—	1.5	0	0.2	0.8	Gaussian
	Cw	cm	0.05	0	0.02	0.025	Gaussian
	Cm	g.cm^-^^2^	0.02	0	0.01	0.01	Gaussian
Canopy	LAI	m^2^/m^2^	7	0	3.5	2.5	Gaussian
	ALIA	degree	80	30	60	20	Gaussian
	hspot	—	1	0	0.45	0.6	Gaussian
Soil	psoil	—	1	0	0.5	0.5	Gaussian
Solar and Sensor	tts	degree	70	25	—	—	Fixed
	tto	degree	80	0	—	—	Fixed
	psi	degree	120	-120	—	—	Fixed
